# Simulation in the clinical setting: towards a standard lexicon

**DOI:** 10.1186/s41077-017-0050-5

**Published:** 2017-09-20

**Authors:** Glenn D. Posner, Marcia L. Clark, Vincent J. Grant

**Affiliations:** 1 Department of Innovation in Medical Education, University of Ottawa, Ottawa, Ontario Canada; 2 Department of Obstetrics and Gynecology, University of Ottawa, Ottawa, Ontario Canada; 3 University of Ottawa Skills and Simulation Centre and The Ottawa Hospital Simulation Patient Safety Program, Ottawa, Ontario Canada; 4 Department of Surgery, Cumming School of Medicine, University of Calgary, Calgary, Alberta Canada; 5 Advanced Technical and Simulation Skills Lab (ATSSL), Calgary, Alberta Canada; 6 Department of Pediatrics, Cumming School of Medicine, University of Calgary, Calgary, Alberta Canada; 7 Department of Emergency Medicine, Cumming School of Medicine, University of Calgary, Calgary, Alberta Canada; 8 KidSIM Simulation Program, Alberta Children’s Hospital, Calgary, Alberta Canada

## Abstract

Simulation-based educational activities are happening in the clinical environment but are not all uniform in terms of their objectives, delivery, or outputs. While these activities all provide an opportunity for individual and team training, nuances in the location, timing, notification, and participants impact the potential outcomes of these sessions and objectives achieved. In light of this, there are actually many different types of simulation-based activity that occur in the clinical environment, which has previously all been grouped together as “in situ” simulation. However, what truly defines in situ simulation is how the clinical environment responds in its’ natural state, including the personnel, equipment, and systems responsible for care in that environment. Beyond individual and team skill sets, there are threats to patient safety or quality patient care that result from challenges with equipment, processes, or system breakdowns. These have been labeled “latent safety threats.” We submit that the opportunity for discovery of latent safety threats is what defines in situ simulation and truly differentiates it from what would be more rightfully called “on-site” simulation. The distinction between the two is highlighted in this article, as well as some of the various sub-types of in situ simulation.

As simulation-based education (SBE) has become more commonplace throughout our healthcare system, a more precise and shared vocabulary amongst simulationists has become essential. The recent release of the Healthcare Simulation Dictionary by the Society for Simulation in Healthcare [1], as well as other published reviews, have been invaluable in establishing a common lexicon and taxonomy for SBE [2]. One of the emerging themes within SBE is the importance of context and the physical setting of SBE, especially SBE that is taking place in or near the clinical setting [3]. Traditionally, SBE occurred separate from the clinical context in simulation centers, termed “off-site simulation” [3]. Over the past decade, the *place* in which SBE occurs has adapted to include on-site clinical spaces. The rationale for this are numerous, but the main impetus for moving SBE into the workplace generally relate to convenience of access for healthcare workers and the ability to train real teams of professionals who commonly work together [4]. This form of SBE has been universally named in situ simulation (ISS). As it pertains to SBE, the traditional definition of ISS describes simulation-based activities that take place in the actual context in which clinical care is being provided [1]. However, from our experience, not all ISS sessions are uniform. There are several nuances and variations within the term “in situ,” each having its own strengths and weaknesses. We believe that it has become important to further define some of the common terms used to describe the setting in which SBE is taking place. In this commentary, these differences will be presented and placed in context, with the goal of advocating for a common lexicon for ISS and further assisting simulationists in matching ISS activities to desired objectives and outcomes.

Although the participants in our examples involve interprofessional teams of healthcare providers (nurses, physicians, respiratory therapists, radiology technicians, pharmacists, trainees at all levels, etc.), ISS can truly be used to teach or assess participants at any level from any vocation either individually or in teams based on clear objectives.

## Form follows function

The goal of most SBE activities in healthcare is to train providers, as either individuals or in interprofessional or multidisciplinary teams, in the proper assessment and management of clinical problems and the practice of psychomotor skills, through deliberate practice and reflective debriefing in a surrogate setting where actual patients will not be harmed [4]. When these activities take place at a simulation center, attempts are made to recreate the clinical setting through attention to physical, conceptual, and emotional realism [5, 6] but they remain, by definition, not real clinical settings. When simulation-based activities take place in the actual clinical setting, they are collectively referred to as *ISS*. While these activities are often challenging to coordinate, they have the added benefit of adding an element of physical and contextual realism that is missing at the simulation center. As an example, we have noted that an oft-stated impediment to engagement in SBE is when the team or participant disengages during the debriefing, rejecting the fiction contract, and claiming that their performance would have been different had they been in their real clinical setting with their real equipment and real colleagues as team members. However, beyond the additional elements of realism that benefit ISS is the invaluable opportunity to also assess and address system- and process-related issues that interact with provider care in the clinical environment. These latent safety threats (LSTs) are defined as threats to patient safety or quality care that result from challenges with equipment, processes, or system breakdowns [7]. As the value of SBE and how it impacts patient outcomes continues to evolve, a modality originally used to create opportunities for learning in a safe environment is now being used to discover threats to patient safety that might otherwise go undetected until a critical incident occurs [8]. The discovery and mitigation of LSTs in an organization can be a powerful and tangible outcome measure that is of significant value to stakeholders, and these opportunities should be seized. We submit that this opportunity for discovery of LSTs is what truly defines ISS and differentiates it from what would be more accurately called “on-site” simulation. The important distinction between these two sub-types of simulation in the clinical setting are highlighted below, as well as various sub-types of ISS.

As we begin to discuss the lexicon of ISS, imagine that you are a simulationist asked to design an ISS for the emergency department and consider these two scenarios:

1. Vignette #1
  A group of emergency room nurses and physicians assemble in an unused area of the emergency department for a team-training exercise involving a simulated case. During the pre-briefing, they are instructed to treat the mannequin as though she was a real patient, they are oriented to a cart of equipment dedicated for this session and a tray of mock medications, and they are told that they can ask for help but that no external codes will be activated throughout the hospital. When the scenario begins, the patient is found to be a pregnant woman near term who has been in a motor vehicle accident. The team manages the patient appropriately, performing a primary survey, assessing the fetal heart rate, and indicating that they would want to call the trauma team and consult obstetrics. The patient suddenly suffers a significant clinical deterioration, and the team prepares for an emergency cesarean section while performing CPR. Afterwards, during the debriefing, the instructors provide feedback regarding team dynamics and crisis resource management principles, as well as reviewing the management of trauma in pregnancy.
2. Vignette #2
  A group of healthcare providers working in the emergency department are told that a simulation-based exercise will be conducted during their shift today and they should manage the situation as they normally would. During the shift, they are called to the resuscitation room where they encounter an obstetrical mannequin. They are told that a pregnant woman near term has been in a motor vehicle accident. The team manages the patient appropriately, performing a primary survey, assessing the fetal heart rate, and then activating the trauma and obstetrics teams. As the trauma team arrives, the patient suddenly suffers a significant clinical deterioration and the team prepares for an emergency cesarean section while performing CPR. Afterwards, during the debriefing, the instructors provide feedback regarding team dynamics and crisis resource management principles, as well as reviewing the management of trauma in pregnancy. The team expresses concerns regarding how long it took for the obstetrician to be located and wonder if they should have activated the obstetrical emergency code, even though the patient was still stable. They note that the perimortem cesarean tray was hard to locate in the department and that it should probably be clearly labeled and closer to the trauma bay. They also found that the suction apparatus in that resuscitation room was not functioning correctly. This information is noted by a clinical manager, and these issues are addressed.

### On-site vs. in situ simulation

Vignette #1 describes an on-site simulation, performed in a repurposed (or unused) room in the emergency department for convenience. It can be challenging to assemble a real clinical team at the simulation center, especially at a location that is off-site. Geography and time collude to prevent the healthcare team from escaping the hospital and convening at the simulation center for “ex situ” training exercises. Healthcare trainees have a structured curriculum and protected time away from clinical responsibilities that somewhat overcome this barrier. However, as we come to value the role of simulation in interprofessional education, the barrier of geography and time for other team members often prevents this training from occurring in the simulation center. In addition, as we further value the role of SBE for continuing professional development [9], more and more independent healthcare practitioners will be involved in SBE and getting them to the simulation center at the same time will continue to be difficult. Furthermore, simulation centers do not exist everywhere, or they do not have the capacity to host the complete array of SBE that is required in their communities.

Many programs have chosen to overcome this barrier by either transporting their equipment (e.g., task trainers, mannequins) to or housing their equipment at the hospital, and conducting SBE in non-clinical spaces, or underused or repurposed clinical areas, often near the real clinical setting. They may create a dedicated space for SBE that is in close proximity to the clinical setting and physically resembles the space they are emulating. They may bring in staff on a volunteer basis or on-duty staff who are temporarily relieved of their clinical duty to participate in these sessions. They may also use equipment that is earmarked for simulation, including “dummy” or “pretend” medications. In these sessions, the real clinical activity is happening in parallel nearby, and in some cases, the real code team or the real clinicians and providers on-call or working in that area are not disturbed. These “peri-situ” (term coined by David Gaba, MD, in 2015) or on-site simulation-based team training exercises are useful for practicing clinical events and discussing team issues. The potential advantages of on-site simulation is that the real clinical space is not disturbed in case it is urgently needed by a real patient, the healthcare professionals currently on-shift are not distracted from their tasks, and real equipment and potentially costly medication is not damaged or wasted. Furthermore, by keeping the case local, the session is easier to coordinate and less disruptive to the entire hospital. On-site simulation might identify latent safety threats related to individual or team knowledge, clinical skills, and behaviors but does not truly audit the system, equipment, and team that will actually be used if the same predicament should befall a similar patient. In this example, had the case been facilitated with the “working” team, using their own equipment in the same environment as a “real” patient, the system could then be audited in its’ entirety and LSTs that exist in that environment could be identified. This does not mean that ISS is necessarily better than on-site simulation, but it is different, and these differences in objectives and anticipated outcomes should be appreciated (Table 1).

**Table 1:** The locations of SBE sessions and the objectives they are designed to address

Objectives	IPE	Latent safety threats identified		
Modality	Team training/CRM skills	Training and knowledge	Maintenance and equipment	Systems and process
Simulation center (ex situ)	+	+	−	−
In situ	+	+	+	+
On-site	+	+	+/−	−

*IPE* interprofessional education

### In situ simulation: audit, education, or both?

Vignette #2 describes an ISS that takes place in the actual clinical setting, with the teams that are currently on-shift for multiple services throughout the hospital. While these sessions provide both education and an opportunity to audit the system, they are logistically more challenging to coordinate, and more disruptive to the local clinical department and potentially the rest of the institution. Furthermore, one must always be cognizant of the unintended consequences of one’s choice of modality because of safety concerns (i.e., cancelation in the face of heavy clinical volume and “dummy” medication being left inadvertently in a clinical setting) [10].

**Fig. 1:**
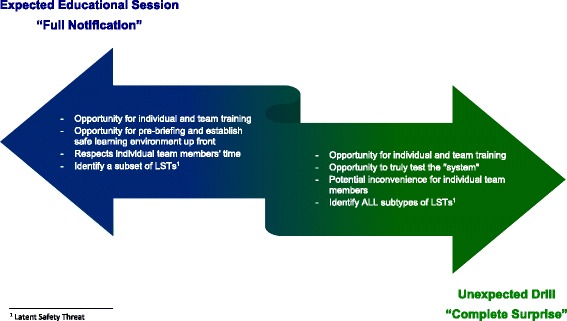
The “expectation spectrum” of in situ simulation

Even within the spectrum of ISS, there are two extremes related to the element of notification (i.e., surprise) involved in these simulation sessions (Fig. 1). In some circumstances, a mannequin is brought to the clinical area and a mock resuscitation code (i.e., “code blue”) is initiated. In these sessions, the participants are generally unaware that a mock code is being called at that time and respond as they would to a real emergency situation (what Sorensen et al. [3] refer to as a “drill”). These sessions not only are conducted for the benefit of team training but also act as an audit of the system. At the other end of the spectrum is formal SBE that is performed in the clinical environment that is well advertised, pre-briefed, and attended by a pre-selected group of individuals (we will call these sessions “expected”). These sessions can also detect some LSTs when they are conducted in the actual clinical setting with the actual local equipment, but the element of surprise is absent. The advantage of the unexpected drill is the assessment of the system response, including response time, staggered leadership assignment, and an evaluation of the functioning of the healthcare team in real time. The disadvantage of the unexpected drill is that people may feel aggravated when pulled from real clinical care to manage a mock situation. Acceptance of this approach requires buy-in and support from both participants and administration. Conversely, the advantage of the expected teaching session is the ability to establish a safe-learning environment through proper pre-briefing and sharing of goals for the session [11], allowing for introductions and questions, as well as demonstrating respect for people’s time. The disadvantage is that certain LSTs (e.g., response time and human resource allocation) cannot be evaluated. There are no implied hierarchy in this taxonomy and no “best” method; the choice of which form of ISS needs to be aligned with the objectives and desired outcomes of the session. Of course, an audit can be educational and surprise is not required for identifying some LSTs, so sessions can run the gamut along this continuum. An appreciation for the strengths and weaknesses of these methods, and deciding when to use them appropriately, is essential.

### Just-in-time training and anticipatory simulation

Another sub-type of in situ SBE lies in practicing not “what might happen someday” but “what might happen today” or “what will happen today”. We will refer to these sessions as “just-in-time (JIT) training” and “anticipatory simulation,” respectively. JIT training is “an educational strategy where training occurs in close temporal proximity to a clinical encounter” [12]. Examples include a surgeon who practices a difficult surgical procedure on a digital reconstruction or 3D printed representation of an actual patient’s pathology or an entire healthcare team who practices the motions that will be required in the actual operating theater where a complicated case will soon take place. There are a myriad of applications, but this type of training takes place just prior to an actual clinical event is scheduled to occur. The goal of these just-in-time sessions is to detect LSTs and mitigate risk to the patient by having the team rehearse in advance of a known and potentially complicated maneuver—a “dress rehearsal” as it were.

Anticipatory simulation is similar to JIT training, but instead of practicing a maneuver that *will* happen soon, simulation educators peruse a given ward or clinical area and determine what *might* happen soon: “What is the worst thing that could happen on this ward overnight? Which patient is most likely to deteriorate and what would that deterioration be? What is the most likely complication that could happen to this patient today, and can the actual team practice that scenario nearby so that they are better prepared to manage it if it happens?” The goal of such anticipatory SBE sessions is very similar to pure in situ simulation in that they identify LSTs and mitigate them before they possibly happen for real. The subtle difference between the two sub-types lies in the *needs assessment* that drives scenario selection. In the former, this is based on the anticipated needs of current patients in the clinical milieu as opposed to the latter, which is often based on abstract objectives of training, recent safety incidents, a clinical early warning score, or perceived discomfort with crises amongst the potential participants. Another value of anticipatory simulation is that it is grounded in the possible and the participants understand the direct relevance of the exercise to their patient care, creating better buy-in that this training is worthwhile. At a recent perimortem cesarean section that took place in the emergency department, a healthcare provider exclaimed, “Hey everyone, let’s just do it the same way we did it this morning during sim!”

### Towards a standard lexicon

**Fig. 2:**
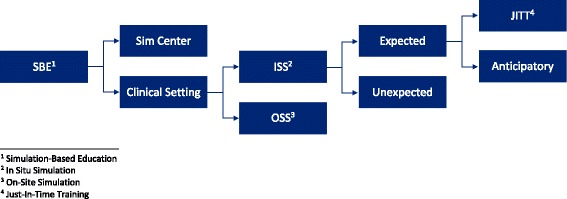
The types and subtypes of simulation in the clinical setting

In summary, we anticipate that SBE in the clinical setting will become more and more pervasive, and a common lexicon to describe the various forms of in situ or on-site simulation is needed (Fig. 2). The intention is not to put a value judgment on the variety of simulation taking place in the clinical setting but to choose, based on the goals, objectives and anticipated outcomes of a simulation program, the one that best fits these needs and leverages the strengths of this powerful teaching modality.
